# Communication: Charge transfer dominates over proton transfer in the reaction of nitric acid with gas-phase hydrated electrons

**DOI:** 10.1063/1.4999392

**Published:** 2017-09-14

**Authors:** Jozef Lengyel, Jakub Med, Petr Slavíček, Martin K. Beyer

**Affiliations:** 1 Institut für Ionenphysik und Angewandte Physik, Universität Innsbruck, Technikerstraße 25, 6020 Innsbruck, Austria; 2 Department of Physical Chemistry, University of Chemistry and Technology Prague, Technická 5, 16628 Prague, Czech Republic

## Abstract

The reaction of HNO_3_ with hydrated electrons (H_2_O)_*n*_^−^ (*n* = 35–65) in the gas phase was studied using Fourier transform ion cyclotron resonance (FT-ICR) mass spectrometry and *ab initio* molecular dynamics simulations. Kinetic analysis of the experimental data shows that OH^−^(H_2_O)_*m*_ is formed primarily via a reaction of the hydrated electron with HNO_3_ inside the cluster, while proton transfer is not observed and NO_3_^−^(H_2_O)_*m*_ is just a secondary product. The reaction enthalpy was determined using nanocalorimetry, revealing a quite exothermic charge transfer with −241 ± 69 kJ mol^−1^. *Ab initio* molecular dynamics simulations indicate that proton transfer is an allowed reaction pathway, but the overall thermochemistry favors charge transfer.

Charge transfer (CT) and proton transfer (PT) reactions form the basis for many important processes in chemistry, biology, and technology.^1–4^ The two processes can proceed independently, consecutively, or in parallel as proton coupled electron transfer.^5,6^ PT and CT processes can also compete: once proton transfer has taken place, electron transfer is no longer possible, and vice versa. Here we study the competition between PT and CT for the reaction of hydrated electrons in finite clusters (H_2_O)_*n*_^−^ with gaseous nitric acid. Gas-phase hydrated electrons are quite convenient for such an exploration. First, the finite size allows for a direct observation of various molecular fragments formed in the reactions by mass spectrometry. Second, the energetics of the reaction can be probed using the concept of nanocalorimetry,^7–9^ i.e., by detecting the number of evaporating water molecules when the reaction takes place.

The analogous reaction of (H_2_O)_*n*_^−^ with HCl was studied by Siu *et al.* ^10^ As HCl is a very strong acid, proton transfer prevails. Upon uptake of HCl by the (H_2_O)_*n*_^−^ clusters (*n* = 30–70), HCl dissociates, and the electron recombines with the proton. The nascent H atom evaporates from the cluster and Cl^−^(H_2_O)_*n*_ is observed in the mass spectrum. Since HNO_3_ also is a strong acid, one might expect that the reaction of HNO_3_ with hydrated electrons should result in NO_3_^−^(H_2_O)_*n*_. On the other hand, HNO_3_ is a slightly weaker acid than HCl and at the same time it readily undergoes dissociative electron transfer in the gas phase.^11–13^ Charge transfer leading to NO_2_^−^(H_2_O)_*n*_ or OH^−^(H_2_O)_*n*_ is therefore also conceivable. In fact, all three potential product species, NO_3_^−^(H_2_O)_*n*_, NO_2_^−^(H_2_O)_*n*_, and OH^−^(H_2_O)_*n*_, have been observed in our recent study,^14^ where low-energy free electrons were brought to interact with neutral mixed nitric acid–water clusters (HNO_3_)_*m*_(H_2_O)_*n*_, *m* ≈ 1–6, *n* ≈ 1–15.

The mechanism of the gas phase reaction between free electrons and HNO_3_ was studied in detail using flowing afterglow techniques. Dissociative electron attachment to HNO_3_ yields primarily NO_2_^−^ in a very efficient exothermic process with an energy release of around 13 kJ mol^-1^.^11–14^ Shuman *et al.* ^13^ observed the formation of OH^−^ as a minor channel, which is 30 kJ mol^- 1^ endothermic. The formation of NO_3_^−^ in the gas phase is even more endothermic with 43 kJ mol^- 1^ and has recently been observed using a crossed-beam experiment.^14^ However, electron driven processes often dramatically change upon solvation.^15–18^ Hydration affects the electronic structure of transient negative ions and enhances or suppresses reaction channels. Furthermore, HNO_3_ has a strong affinity to ice,^19^ where it rapidly dissociates.^20–22^

To experimentally resolve these issues, we studied the reaction of HNO_3_ with (H_2_O)_*n*_^−^ (*n* = 35–65) by Fourier transform ion cyclotron resonance (FT-ICR) mass spectrometry. The measurements are complemented with *ab initio* molecular dynamics simulations.

Figure 1 shows mass spectra of the reaction of HNO_3_ with (H_2_O)_*n*_^−^ at characteristic reaction delays. The reaction results in two intense product ions: OH^−^(H_2_O)_*n*_ and NO_3_^−^(H_2_O)_*n*_. In addition, a small amount of NO_2_^−^(H_2_O)_*n*_ is observed. At *t* = 0 s, [Fig. 1(a)], the mass spectrum is dominated by hydrated electrons. However, a significant amount of OH^−^(H_2_O)_*n*_ as well as traces of NO_3_^−^(H_2_O)_*n*_ is present due to reactive collisions during the ion accumulation in the ICR cell, which takes 2 s. At *t* = 3 s, Fig. 1(b), roughly equal amounts of (H_2_O)_*n*_^−^ and OH^−^(H_2_O)_*n*_ are present, and NO_3_^−^(H_2_O)_*n*_ is catching up. The strong increase of the NO_3_^−^(H_2_O)_*n*_ intensity between 0 s and 3 s indicates that NO_3_^−^(H_2_O)_*n*_ is formed as a secondary product. At longer times [Fig. 1(c)], multiple pick-up of HNO_3_ and the significant effect of blackbody infrared radiative dissociation (BIRD)^23^ are observed. This leads to complete water evaporation and the formation of NO_3_^−^HNO_3_ and NO_3_^−^(HNO_3_)_2_ cluster ions. Analogous cluster ions were found as final products in gas-phase ion-molecule reactions^11,24–26^ and electrospray ionization of aqueous HNO_3_ solution.^27^

Our kinetic analysis assuming pseudo-first order kinetics, Fig. 2(a), confirms unambiguously that hydrated electrons react exclusively to OH^−^(H_2_O)_*n*_, reaction (1), while NO_3_^−^(H_2_O)_*n*_ clusters are formed as a secondary product, reaction (2). The perfect pseudo-first order behavior also indicates that the reaction rate is independent of the cluster size. Obviously, charge transfer to HNO_3_ followed by dissociation is faster than the acidic dissociation of HNO_3_, which would lead to hydrogen formation analogous to the HCl reaction. Since a mixture of HNO_3_ and H_2_O vapor is present in the ICR cell, we cannot derive a reliable pressure-independent rate constant. Using the total measured pressure as the partial pressure of HNO_3_, we obtain lower limits for the rate constants *k*(1) ≥ (2.8 ± 1.1) × 10^−10^cm^3^ s^−1^ and *k*(2) ≥ (2.4 ± 0.9) × 10^−10^cm^3^ s^-1^, while the upper limits are given by the collision rates,

(1) $${{\left({\text{H}}_{2}\text{O}\right)}_{n}}^{-}+{\text{HNO}}_{3}\to {\text{OH}}^{-}{\left({\text{H}}_{2}\text{O}\right)}_{m}+{\text{NO}}_{2}+(n-m){\text{H}}_{2}\text{O},$$

(2) $${\left({\text{H}}_{2}\text{O}\right)}_{m}{\text{OH}}^{-}+{\text{HNO}}_{3}\to {{\text{NO}}_{3}}^{-}{\left({\text{H}}_{2}\text{O}\right)}_{l}+(m-l+1){\text{H}}_{2}\text{O}.$$

Reaction (2) is the well-known acid–base reaction.^26,28^ The NO_3_^−^ anion is often seen as a terminal product in many ion-molecule reactions involving HNO_3_^11,26,29^ and also occurs naturally in the troposphere.^30^ NO_3_^−^(H_2_O)_*n*_ undergoes ligand exchange reactions with additional HNO_3_ molecules resulting in the formation of mixed NO_3_^−^(HNO_3_)_1-4_(H_2_O)_*k*_ cluster ions.

The minor product series NO_2_^−^(H_2_O)_*n*_ may be formed via reaction (3) in competition with reaction (1). However, the presence of traces of HONO as a decomposition product of HNO_3_ on the apparatus walls has to be taken into account, which would afford reactions (4) and (5). Unfortunately, the kinetic fits are ambiguous, due to the low intensity of this product. However, the overall shape of the kinetics curve over all six different experiments is most consistent with NO_2_^−^(H_2_O)_*n*_ formation in the second reaction step, i.e., reaction (5),

(3) $${{\left({\text{H}}_{2}\text{O}\right)}_{n}}^{-}+{\text{HNO}}_{3}\to {{\text{NO}}_{2}}^{-}{\left({\text{H}}_{2}\text{O}\right)}_{m}+\text{OH}+(n-m){\text{H}}_{2}\text{O},$$

(4) $${{\left({\text{H}}_{2}\text{O}\right)}_{n}}^{-}+\text{HONO}\to {{\text{NO}}_{2}}^{-}{\left({\text{H}}_{2}\text{O}\right)}_{m}+\text{H}+(n-m){\text{H}}_{2}\text{O},$$

(5) $${\left({\text{H}}_{2}\text{O}\right)}_{m}{\text{OH}}^{-}+\text{HONO}\to {\left({\text{H}}_{2}\text{O}\right)}_{l}{{\text{NO}}_{2}}^{-}+(m-l+1){\text{H}}_{2}\text{O}.$$

The plot of average cluster sizes as a function of time, Fig. 2(b), shows that the OH^−^(H_2_O)_*m*_ ion distribution is significantly shifted to smaller cluster sizes relative to that of the hydrated electrons (H_2_O)_*n*_^−^. The loss of water molecules indicates an exothermic reaction. We therefore applied the nanocalorimetry approach, in which the exothermicity of the reaction is determined via the average number of evaporated water molecules.^7–9^ The mean cluster sizes for reactants and products as well as their difference are plotted as a function of time, Figs. 2(b) and 2(c), and fitted with a set of differential equations that account for the water loss due to reaction as well as BIRD.^23^ Note that the time dependence of the difference in Fig. 2(c) is due to a complex interplay of BIRD, reaction kinetics and the 2 s long fill cycle of the cell. Since the product ions present at 0 s arise from ions residing for longer times in the cell, they are smaller than expected, and the difference seems artificially large. As shown before, the differential equations used for the fit describe these effects faithfully.^8^ A nanocalorimetric fit reveals a result of 5.7 ± 1.6 and 2.3 ± 0.2 evaporated water molecules for reactions (1) and (2), respectively. With the energy required to evaporate a single water molecule from the cluster, Δ*E*_vap_ = 43.3 ± 3.1 kJ mol^- 1^,^31,32^ and thermal corrections as detailed in the supplementary material,^8,33,34^ this translates to Δ_r_ *H*_exp_(298 K) =-241 ± 69 kJ mol^-1^ for reaction (1) and -94 ± 11 kJ mol^-1^ for reaction (2).

Table I summarizes the measured reaction enthalpy in comparison with *ab initio* calculation and literature thermochemical data from bulk aqueous solution. The measured energy release of the CT reaction agrees within error limits with the literature value of the room temperature reaction enthalpy in the condensed phase. The thermochemistry also makes it plausible that no NO_3_^−^ formation is observed in the first reaction step. The bulk value for the formation of NO_2_^−^, reaction (3), is only slightly less exothermic than for reaction (1), which would be consistent with its occurrence as a minor primary reaction pathway, as well as with the average cluster size of NO_2_^−^(H_2_O)_*m*_ [Fig. 2(b)]. However, since we know from other experiments^14,29^ that traces of HONO are inevitably present in the reactant gas, formation of NO_2_^−^(H_2_O)_*m*_ is most likely due to collisions with HONO.

To get a mechanistic understanding of the primary reaction, we performed *ab initio* molecular dynamics simulations on small model systems. We started the simulations with equilibrated hydrated electrons with 15 water molecules, where the vertical detachment energy (VDE) is above 1 eV.^38^ The calculated values are slightly larger than the measured data for somewhat larger finite size clusters (see the supplementary material for details).^39^ Then we let react a neutral HNO_3_ molecule placed randomly at a distance of 7.5 Å from the center of mass of the water cluster. Figure 3 shows the evolution of quantities characterizing structures and charge distribution along two selected MD trajectories. Panel A displays a trajectory in which the CT takes place and the non-planar radical anion of nitric acid is formed. The vertical ionization energy of the isolated anionic water oscillates above 1 eV while the (adiabatic) electron affinity for HNO_3_ was measured to be 0.6 eV.^40,41^ The charge transfer reaction is facilitated by solvation of the nitric acid molecule. Indeed, the CT is exothermic for larger clusters (see the supplementary material), yet an energy barrier is expected for this process. In our simulations, the CT reaction was typically observed in tens of picoseconds after the HNO_3_ molecule and the anionic cluster get in contact.

The nascent HNO_3_^−^ dissociates (again on the picosecond time scale) upon interaction with water. For about 1 ps, the negative charge is localized on the NO_2_ moiety. Unlike for the unsolvated reaction, the dissociation into NO_2_^−^ is not observed in the MD simulations. In the presence of a hydration shell, the electron is instead transferred from NO_2_^−^ to OH, yielding OH^−^ and NO_2_. Interestingly, dissociation of the N–O bond takes place concomitantly with charge localization on the OH moiety, and the oscillations in the charge distribution are mirrored in the oscillations of the N–O distance. The NO_2_ molecule then typically leaves the cluster, [reaction (1)], in line with the experimentally observed reaction (1). Although not observed in the trajectory calculations, the relatively long localization of the negative charge on the NO_2_ moiety suggests that the formation of NO_2_^−^(H_2_O)_*m*_ via reaction (3), which would require N–O bond cleavage and OH evaporation before charge transfer from NO_2_^−^ to OH can take place, is a plausible scenario. The difference in thermochemistry between the two pathways is relatively small, therefore the detailed hydration environment of the two product species will be crucial for ultimate charge localization. Minor changes in the hydrogen-bonded network may thus favor one or the other. Besides bond breaking, evaporation of water molecules was observed in some trajectories already on the picosecond time scale.

The simulations, however, also yield trajectories where the proton transfer takes place first and, subsequently, the hydrated electron recombines with H_3_O^+^, leading to H_2_O + H units, with the H atom leaving the cluster,

(6) $${{\left({\text{H}}_{2}\text{O}\right)}_{n}}^{-}+{\text{HNO}}_{3}\to {\left({\text{H}}_{2}\text{O}\right)}_{m}{{\text{NO}}_{3}}^{-}+\text{H}+(n-m){\text{H}}_{2}\text{O}.$$

The trajectory presented in Fig. 1(b) displays a proton transfer taking place in 1.5 ps. The proton then hops several times before the H_3_O^+^ accepts the electron and forms free neutral hydrogen at about 8 ps. In this particular case, the hydrogen atom remains trapped in the cluster for another 2.5 ps before it leaves.

Altogether, we have performed 25 simulations lasting up to 25 ps [the simulations were stopped once either the reaction (1) or (6) took place]. Within that time, we have seen 16 times charge transfer (in 10 cases, the reaction was followed by the subsequent decomposition reaction within the 25 ps time window) and 6 times the proton transfer reaction. No reactive event occurred within the first 25 ps in the three remaining trajectories. We thus observe that both the CT and PT processes are very fast; the fast PT is consistent with previous studies on HNO_3_ dissociation.^42^ We can safely conclude that both the CT and PT channels are ultrafast processes, i.e., the reaction rate is controlled by the collision rate between the nitric acid and anionic cluster. The exact branching ratio is however beyond the scope of the *ab initio* dynamics based on DFT methods. In fact, we know that the vertical detachment energies of the hydrated electron are overestimated in our MD simulations based on the BLYP functional (see the benchmark calculations in the supplementary material). The higher VDEs in a Marcus theory picture result in a higher activation barrier for the CT process. The calculated yield for the CT process thus represents a lower bound estimate. The reactivity of the hydrated electron depends also on its binding energy, which changes with the cluster size. The cluster sizes used in the experiment are greater than in the simulation to avoid competing electron detachment activated by black-body radiation which occurs in (H_2_O)_*n*_^−^, *n* < 30.^43,44^ Therefore, the experiments were performed with a cluster size distribution that started only above this threshold, however, not tractable for *ab initio* simulations.

Our calculations show that the reaction enthalpy gradually decreases with increasing cluster size (*n* ≤ 15; see the supplementary material, Table S3) and, for reaction (1), it slowly reaches the experimental value. An extrapolation of the total energies to the bulk (Table I) by embedding a small anion water cluster in a dielectric continuum^45^ results in a good agreement with literature thermochemical data from bulk aqueous solution for reactions (1) and (2); see the supplementary material. Note that reaction (2) is much less exothermic and this exothermicity decreases with increasing number of solvating water molecules.

In summary, we have demonstrated that the charge transfer reaction between hydrated electron and HNO_3_ is an ultrafast process taking place on the picosecond time scale in finite-size water particles. The transient negative ion HNO_3_^−^ is formed faster than the ionic dissociation of the acid molecule in the water cluster can occur. The excess electron destabilizes the HO–NO_2_ bond. The competition between OH and NO_2_ for the electron is won by OH due to the high hydration enthalpy of OH^−^.

See supplementary material for experimental and theoretical details; conversion of Δ*E*_raw_ to ΔH_0_; thermochemistry of bulk analogues for reactions (1)–(3) and (6); further calculation details; and benchmark calculations.

## Supplementary Material

Supporting Information

## Figures and Tables

**Fig. 1:**
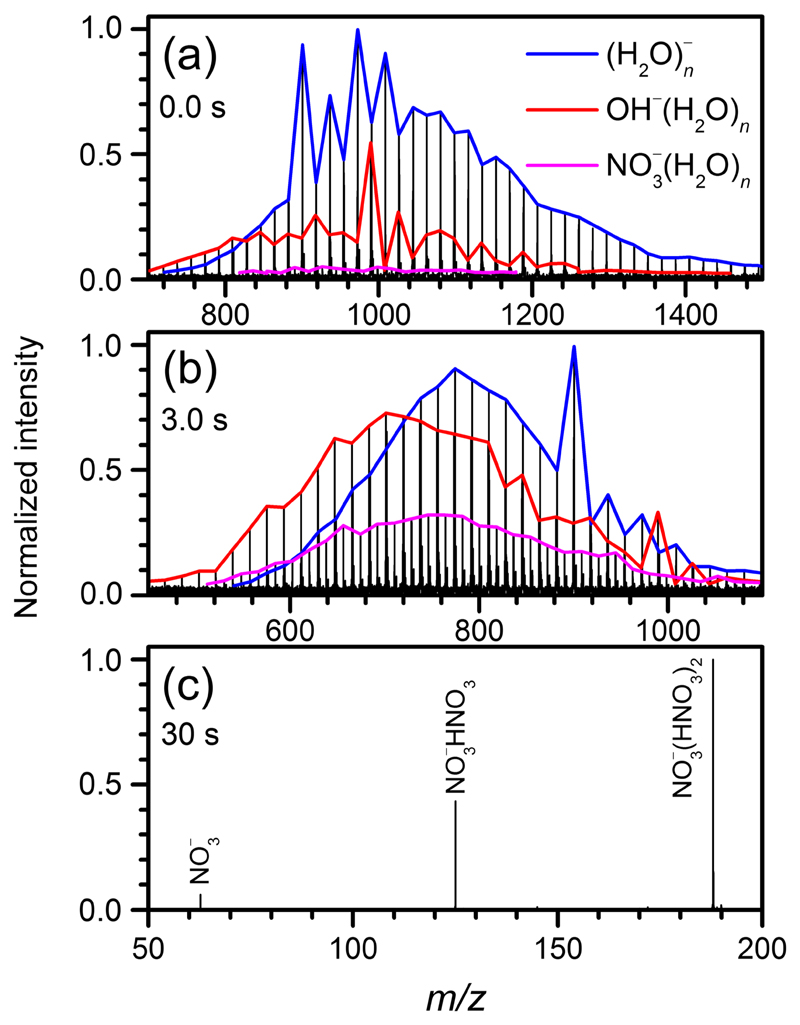
Mass spectra of the reaction of HNO_3_ with hydrated electrons (blue line) after (a) 0.0, (b) 3.0, and (c) 30 s. OH^−^(H_2_O)_*n*_ (red line) and NO_3_^−^(H_2_O)_*n*_ (purple line) are observed as product ions.

**Fig. 2:**
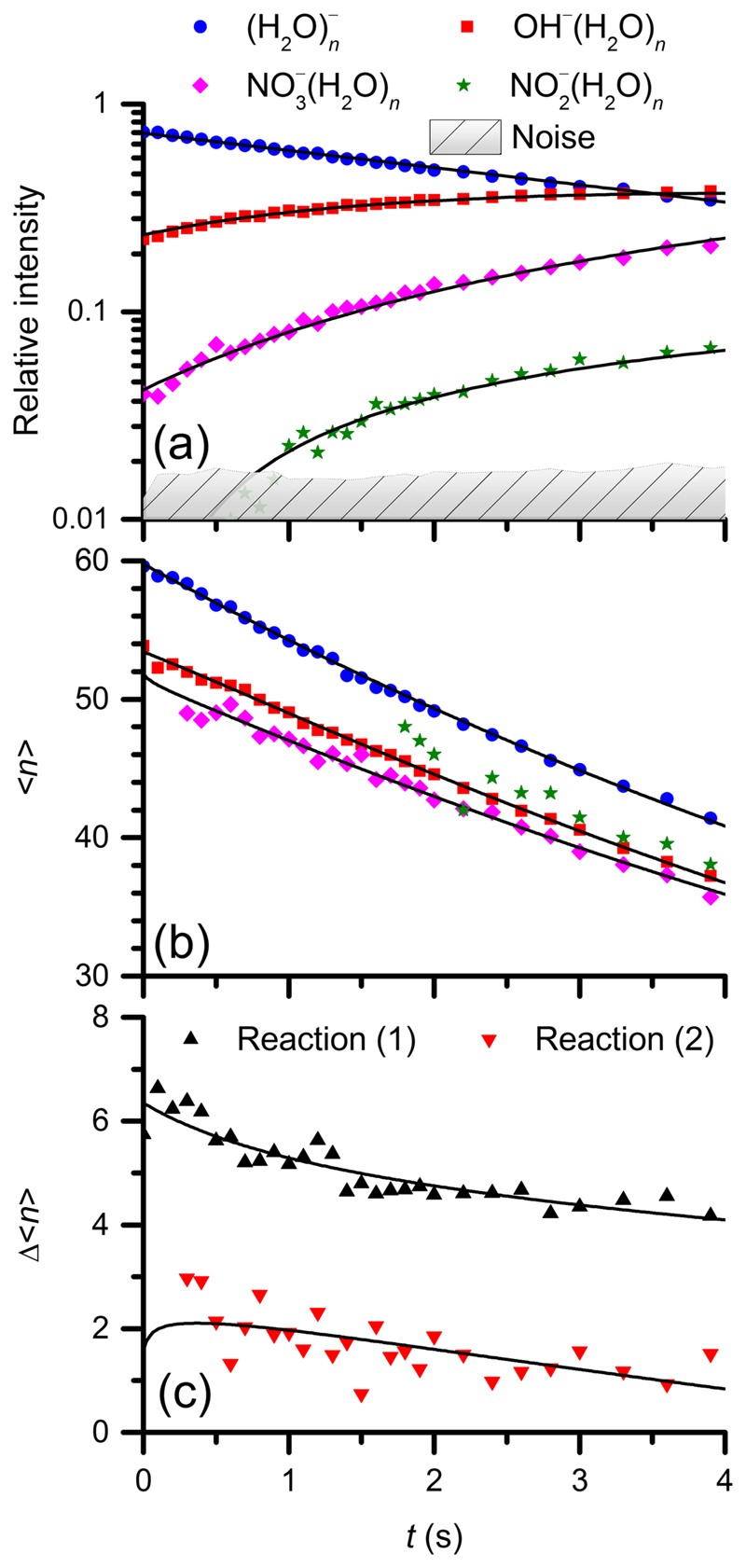
(a) Kinetic and (b) and (c) nanocalorimetric analysis of the reaction of HNO_3_ with hydrated electrons (H_2_O)_*n*_^−^ at room temperature. Panel (a) represents the pseudo-first-order kinetic fit of (H_2_O)_*n*_^−^ (blue circles) as the reactant and OH^−^(H_2_O)_*n*_ (red squares), NO_3_^−^(H_2_O)_*n*_ (purple diamonds), and NO_2_^−^(H_2_O)_*n*_ (green stars) as the product ions. Panel (b) shows the fit of the cluster mean sizes for the reactant and product ions, and panel (c) illustrates the fit of their size difference for reaction (1)[Δ(〈*n*〉e_hyd_^−^ - 〈*n*〉OH^−^); black up triangles] and reaction (2) [Δ〈*n*〉OH^−^ - 〈*n*〉NO_3_^−^); red down triangles].

**Fig. 3:**
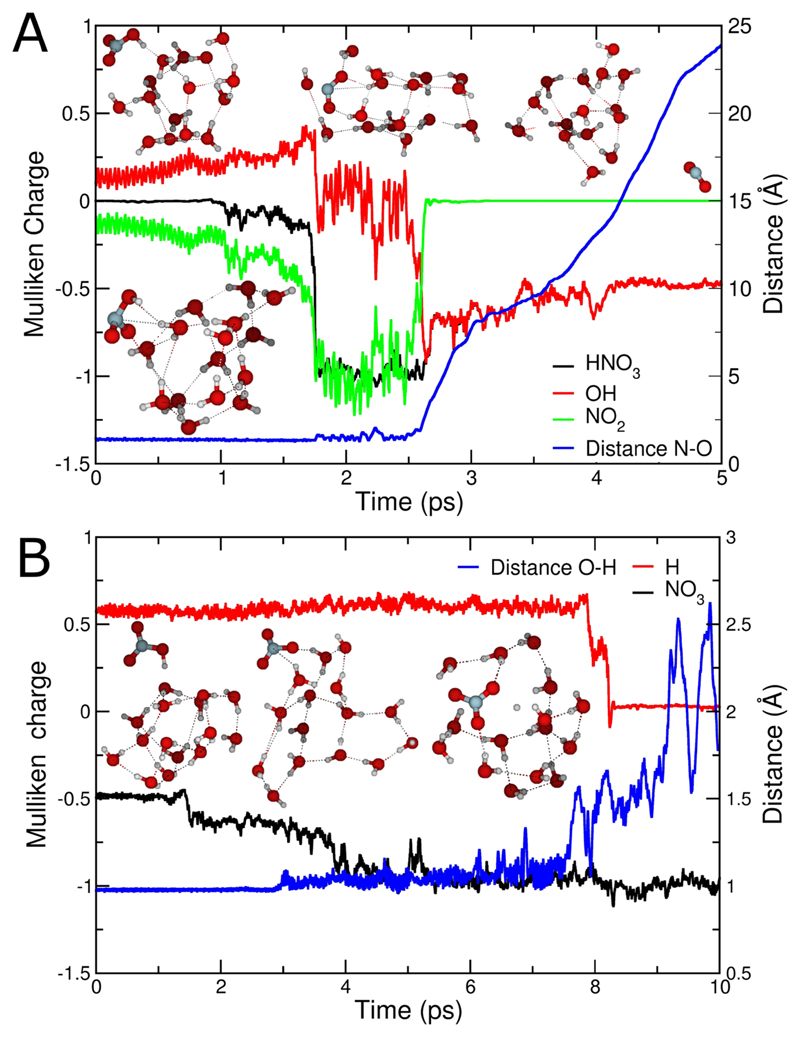
Two representative trajectories for the reaction of HNO_3_ with (H_2_O)_15_^−^. Panel (a) shows reaction (H_2_O)_15_^−^+ HNO_3_ → OH^−^(H_2_O)_15_ + NO_2_. The curves represent Mulliken charges of different moieties (black = HNO_3_, red = OH, and green = NO_2_) and the O-N bond distance in the nitric acid (blue); panel (b) shows reaction (H_2_O)_15_^−^+ HNO_3_ → (H_2_O)_15_NO_3_^−^ + H (black curve = NO_3_^−^, red curve = leaving hydrogen) and the O–H bond distance in the nitric acid (blue).

**Table I:** Reaction energetics for reactions of HNO_3_ with (H_2_O)_*n*_^−^ and OH^−^(H_2_O)_*n*_ in units of kJ mol^-1^.

	Experiment cluster	Experiment bulk	Calculation cluster *n* = 15	Calculation bulk *n* = 6+ PCM
Reaction (1)	- 241 ± 69	-258 ± 11^13,35,36^	- 248 ± 7	-278
Reaction (2)	- 94 ± 11	- 129^37^	- 164 ± 6	- 158
Reaction (3)	…	- 246 ± 11^13,35,36^	- 177 ± 9	- 212
Reaction (6)	…	- 99 ± 11^13,35,36^	- 82 ± 8	- 95
