# Supplementary material for: Communication: Charge transfer dominates over proton transfer in the reaction of nitric acid with gas-phase hydrated electrons
Source: J Chem Phys. Author manuscript; Available in PMC 2020 Nov 6. (PMC7116334; doi:10.1063/1.4999392)
Supplement: Supporting Information [file EMS102278-supplement-Supporting_Information.pdf]

Supplementary material: Charge transfer dominates over proton transfer in the reaction of nitric acid with gas-phase hydrated electrons

Jozef Lengyel,[1,a)] Jakub Med,[2] Petr Slavíček,[2,b)] and Martin K. Beyer[1,c)]

[1] *Institut für Ionenphysik und Angewandte Physik, Universität Innsbruck, Technikerstraße 25, 6020 Innsbruck, Austria.*

[2] *Department of Physical Chemistry, University of Chemistry and Technology Prague, Technická 5, 16628 Prague, Czech Republic*

[a)] Electronic mail: jozef.lengyel@uibk.ac.at
[b)] Electronic mail: petr.slavicek@vscht.cz
[c)] Electronic mail: martin.beyer@uibk.ac.at

1. Methods: Experimental

The experiments are performed on a modified Bruker/Spectrospin CMS47X FT-ICR mass spectrometer, equipped with a 4.7 T superconducting magnet, a Bruker infinity cell, and an APEX III data station.[1,2] Anionic water clusters $(H_2O)_n^-$ are generated in a home built external source[1,2] by laser vaporization of a solid zinc target and seeding the helium carrier gas with traces of H_2O. The skimmed $(H_2O)_n^-$ cluster beam is transferred via an electrostatic lens system through differential pumping stages into the ultra-high vacuum (UHV) region of the mass spectrometer, with a background pressure below 5.0×10^{-10} mbar, and stored in the ICR cell. HNO_3 is introduced into the UHV region from the headspace of a 69% aqueous solution through a gold sealed leak valve at a constant pressure in range of $(1.9\text{-}3.3)\times10^{-8}$ mbar. To reduce the gas-phase impurities concentrated HNO_3 aqueous solutions was used rather than fuming nitric acid, in which a significant amount of $NO_2^{\bullet}$ and other decomposition products is present. Before each experiment, a cold trap to freeze HNO_3 aqueous solution was set up to in order to remove gaseous and highly volatile contaminants from the solution. The purity of the reactant is checked using electron ionization directly in the ICR cell and it is compared to the published data.[3] However, the significant amount of water in solution reduces the measured rate constants of reactions which are lowered compared to the collision rate. For current experiment, this represents around ≈10% of the collision rate.

To determine the rate constant, reactions are monitored by recording mass spectra as a function of time. The intensities of reactant and product ions in the mass spectra are summed over all cluster sizes and normalized. The kinetic fit yielded a pseudo-first-order rate constant (k_{REL} / s^{-1}), which is converted to a pressure corrected absolute rate constant (k_{abs} / $cm^3\ s^{-1}$). A relative error of ±40% is assumed, determined by the uncertainty of the pressure calibration and the water content in the head space of the concentrated HNO_3 solution. Since the gas phase HNO_3 pressure is a critical parameter for the absolute rate constant determination, the measurements are performed at different pressures repeatedly on different days to minimize uncertainties. The reaction kinetics is fitted up to 4 s because blackbody radiation induced electron detachment occurs in below $n = 30$.[4,5]

Thermochemistry is investigated using nanocalorimetry.[2,6] The heat released during the reaction is extracted by quantitative modelling of the average size of reactant and product clusters as a function of time,[2] taking into account blackbody radiation induced dissociation (BIRD).[7] To extract the reaction enthalpy from the mass spectra, the average cluster size of reactant and product species is calculated. The results are fitted with a genetic algorithm with the following differential equations:

$$\mathrm{d}N_{\mathrm{R}} = -k_{\mathrm{f}}(N_{\mathrm{R}} - N_{0,\mathrm{R}})\mathrm{d}t \quad \text{(S1)}$$

$$\mathrm{d}N_{P} = -k_{\mathrm{f}}\left(N_{\mathrm{P}} - N_{0,\mathrm{P}}\right)\mathrm{d}t + \left(N_{\mathrm{R}} - \Delta N_{\mathrm{vap}} - N_{\mathrm{P}}\right)\left(\frac{kI_{\mathrm{R}}}{I_{\mathrm{P}}}\right)\mathrm{d}t \quad \text{(S2)}$$

Eq. (S1) and the first term in eq. (S2) describe BIRD of water clusters, with k_{f} describing the linear dependence on cluster size. $N_{0,\mathrm{R}}$, $N_{0,\mathrm{P}}$ account for the contribution of the ionic core to the IR absorption cross sections. The second term in eq. (S2) describes the evaporation of water molecules due to the reaction enthalpy released in the water cluster. The average number of evaporated water molecules ΔN_{vap} is the key result of the fit.

2. Methods: Computational

The *ab initio* molecular dynamics (MD) simulations were executed with the Terachem quantum chemistry code allowing for a rapid DFT calculation due to the GPU based technology.[8,9] Still, the calculations are relatively time-demanding. The $(H_2O)_{15}^-$ anionic water clusters were therefore simulated at the BLYP/6-31+g* level[10,11] with Grimme's D2 dispersion correction, allowing for efficient calculations.[12] The dispersion correction does not have a major effect on the calculations. As we show below , the electron is relatively strongly bound in larger water anionic clusters so that the relatively small 6-31+g* basis set can be used. The constant temperature of 150 K was maintained with Nosé-Hoover thermostat. The total duration for the simulations was 250 ps, using a timestep of 0.5 fs. The reaction between the HNO_3 and the equilibrated $(H_2O)_{15}^-$ clusters was then modelled with the range-separated functional LC-ωPBE/6-31+g* at constant energy.[13] The course of reaction was monitored by observing geometrical changes, recording Mulliken charges and inspecting spin densities of the unpaired electron. The results are robust with respect to the population analysis used. The energetics for the reactions was evaluated at the hybrid BMK/aug-cc-pVDZ level,[14] providing reliable values of ionization energetics. For small water cluster (with 0 to 6 water units), we have optimized the respective cluster geometries. For the large cluster with 15 water units, we took several snapshots from MD simulation to sample the rich potential energy surface of the system. Further details and benchmark calculations are showed below.

3. Conversion of ΔE_{raw} to $\Delta_r H_{exp}$(298K)

Temperatures in the experiment for reaction:

$(H_2O)_n^-$	+	HNO_3	⟶	$(H_2O)_mOH^-$	+	NO_2	+	$(n-m)\ H_2O$
90-120 K		298 K		90-120 K		120-220 K		

The internal temperature of anionic water clusters is a result of radiative heating and evaporative cooling. We assume a value of 90 – 120 K, which corresponds to the region where the solid-to-liquid phase transition occurs.[15] The neutral reactant is at room temperature, equilibrated in collisions with the surfaces in the UHV region of the mass spectrometer. The neutral products, including the evaporating H_2O molecules, will have an internal energy distribution that corresponds to the internal temperature of the cluster after the reaction. The reaction enthalpy is heating the cluster with 247 kJ mol^{-1}. Setting $n = 50$, there are about $6n = 300$ low-lying vibrational degrees of freedom which correspond to the translational and rotational degrees of freedom of the free water molecules. If we assume that these are thermally populated, we have 0.82 kJ mol^{-1} per degree of freedom. A fully populated vibrational degree of freedom contains RT internal energy, therefore the 0.82 kJ mol^{-1} per degree of freedom correspond to a temperature increase of ≈100 K. Of course, the cluster immediately responds with evaporative cooling, therefore we do not know the exact temperature at which each neutral molecule evaporates. In addition, instead of increasing the temperature, the cluster may convert the additional energy into latent heat by breaking hydrogen bonds. Since a detailed modeling of all these aspects goes beyond the scope of the present work, we give the conservative range above.

From nanocalorimetry, we obtain ΔE_{raw} at these conditions:

$$\Delta E_{raw} = -\Delta_{vap}N\ \Delta_{vap}E = -247 \pm 69 \text{ kJ mol}^{-1}$$

Corrections for $\Delta_r H_{exp}$(298K):

The difference in heat capacity of an anionic water cluster compared to hydrated hydroxide anion is unknown. However, the three vibrational degrees of freedom of the OH^- ion oscillating in the cluster is an upper limit. If these low-lying modes, we have a contribution to the heat capacity of $3R$. Since

the heat of the reaction has to provide the energy to populate these modes, this effect reduces the exothermicity, therefore the correction has a positive sign. Since the correction lies somewhere between 0 and 3 *RT*, we suggest:

$\Delta(\Delta H)\mathrm{C} = (1.5 \pm 1.5)\ [R(298\ \mathrm{K} - 105\ \mathrm{K})] = 2.4 \pm 2.4\ \mathrm{kJ\ mol^{-1}}$

The same argument applies to the neutral reactant and product. For $\Delta_r H_{exp}(298\mathrm{K})$, the NO_2 must be heated to 298 K. The heat capacity of a rigid rotor without thermally populated vibrational levels is 3*R*. The correction therefore amounts to:

$\Delta(\Delta H)\mathrm{NO_2} = 3R\ (298\ \mathrm{K} - (170 \pm 50\ \mathrm{K})] = 3.2 \pm 1.2\ \mathrm{kJ\ mol^{-1}}$

In summary, we obtain with Gaussian error propagation:

$\Delta_r H_{exp}(298\mathrm{K}) = \Delta E_{raw} + \Delta(\Delta H)\mathrm{C} + \Delta(\Delta H)\mathrm{NO_2} = -247+2.4+3.2 \pm \mathrm{sqrt}(69^2+2.4^2+1.2^2)\ \mathrm{kJ\ mol^{-1}} = -241 \pm 69\ \mathrm{kJ\ mol^{-1}}$

4. Thermochemistry of bulk analogues for reactions (1-4)

Table S1. Thermochemistry of bulk analogues for reactions (1-4).

Reaction	$\Delta_r H$(298K) / kJ mol^{-1}
HNO_3(g) + e^-(aq) → OH^-(aq) + $NO_2^\bullet$(g)	–258±11
HNO_3(g) + OH^-(aq) → NO_3^-(aq) + H_2O(l)	–129
HNO_3(g) + e^-(aq) → NO_2^-(aq) + $OH^\bullet$(g)	–246±11
HNO_3(g) + e^-(aq) → NO_3^-(aq) + $H^\bullet$(g)	–99±11

Table S2. Thermochemical cycle for the bulk analogue of reaction (1).

Reaction	$\Delta_r H$(298K) / kJ mol^{-1}	Reference
HNO_3(g) + e^-(g) → OH^-(g) + $NO_2^\bullet$(g)	29.9	[16]
OH^-(g) → OH^-(aq)	–460±10	[17,18]
H^+(aq) + e^-(aq) → H^+(g) + e^-(g)	1261.9±3.8	[19]
H^+(g) → H^+(aq)	–1090	[17,18]
**HNO_3(g) + e^-(aq) → OH^-(aq) + $NO_2^\bullet$(g)**	**–258.2±11**	**sum**

The thermochemical cycles for other reaction pathways (Table S1) were derived using the same approach. $\Delta_r H$(298K) for all individual reactions are available in the above mentioned references.

5. Reaction energetics from DFT calculations

Below we present *ab initio* data on the reaction energetics for the three possible reaction channels considered in our work:

$$(H_2O)_n^- + HNO_3 \rightarrow (H_2O)_n + HNO_3^- \rightarrow OH^-(H_2O)_n + NO_2 \qquad (1)$$

$$OH^-(H_2O)_n + HNO_3 \rightarrow NO_3^-(H_2O)_{n+1} \qquad (2)$$

$$(H_2O)_n^- + HNO_3 \rightarrow (H_2O)_n + HNO_3^- \rightarrow NO_2^-(H_2O)_n + OH \qquad (3)$$

$$(H_2O)_n^- + HNO_3 \rightarrow NO_3^-(H_2O)_n + H \qquad (4)$$

We also present the energy needed for vertical electron transfer from anionic water cluster to nitric acid and the adiabatic energy of the electron transfer. We have performed the calculations for small molecular clusters with less than 6 water molecules. Here, we have used previously observed global minima from literature and re-optimized those geometries at the BMK/-aug-cc-pVDZ level of theory. Geometries from ref. 20 were used as initial structures for the anionic water clusters, geometries from ref. 21 as initial structures for solvated hydroxyl anion clusters and geometries from ref. 22,23 as initial structures for neutral water clusters and ref. 24 for solvated nitric acid anion clusters. Temperature and zero point vibration corrections are not considered. For clusters containing 15 water molecules, we have selected 6 random geometries to sample the potential energy surface. These initial structures were then further re-optimized as above. Reaction energies for n=15 are averaged over 6 randomly sampled structures and the respective error estimate represents a 95% (2σ) confidence interval. We also show the energies of the reaction for the clusters with n=6, embedded in dielectric continuum as implemented with the Polarizable Continuum Model (PCM). These values should be representative of the situation in the bulk.[25] In this case, we have also used geometries of anionic water clusters from Ref. 25 which are designed to mimic energetics of hydrated electron.

The data in the Table S3 points to the level of inaccuracy expected for the present calculations. For example, the energy for adiabatic electron transfer is calculated to be –68 kJ mol^{-1} while the experimental value amounts to −54 kJ mol^{-1}.[27] This value can be refined with electronic structure

methods of higher accuracy, e.g. the CCSD(T)/aug-cc-pVTZ value is –53 kJ mol^{-1} (see *6. Benchmarking the electronic structure calculations*). Such approach is however not feasible for larger clusters.

Table S3. Reaction energetics for different processes related to the interaction of anionic water clusters with HNO_3. All energies in kJ mol^{-1}. For compatibility with vertical electron transfer, equilibrium values are given, without thermal or zero-point corrections.

No. of water molecules	*Vertical electron transfer*	*Adiabatic electron transfer*	*Energy for reaction (1)*	*Energy for reaction (2)*	*Energy for reaction (3)*	*Energy for reaction (4)*
0 (only HNO_3)	28.93	–67.79	63.27	–363.20	–5.20	57.02
1	–46.72	–143.88	–124.78	–305.22	–153.03	–80.50
2	–11.66	–113.75	–165.68	–263.69	–160.57	–86.49
3	10.61	–114.85	–199.96	–240.09	–166.86	–92.37
4	41.82	–114.35	–227.89	–197.72	–182.71	–100.57
5	46.71	–96.10	–216.42	–191.93	–169.87	–80.46
6	60.58	–88.96	–238.45	–183.79	–176.54	–67.67
15 (Ø of 6)	132.47	–73.69	–248.38	–164.45	–176.71	–81.68
6 + PCM	149.84	–214.96	–278.29	–157.57	–211.72	–95.04

6. Benchmarking the electronic structure calculations

The MD simulations were performed with the GGA functional with a relatively limited basis set (BLYP/6-31+g*). This necessarily leads to an error in the calculations rates of the CT and PT processes. To estimate the direction and magnitude of the error for the CT process, we compare the calculated distribution of the VDEs for anionic water clusters with the experiment and with other electronic structure methods. Further, we make the same comparison for vertical attachment energy of the nitric acid. The energetics of these two processes are reflected in the rate of the CT process.

a. $(H_2O)_{15}^{-}$ clusters equilibrating simulations – comparison with experiment

The anionic water clusters $(H_2O)_{15}^{-}$ were equilibrated in 5 independent Molecular Dynamics runs, using *ab initio* molecular dynamics with BLYP functional, 6-31+g* basis set and Grimme's D2 dispersion correction. Each trajectory was equilibrated for 50 ps. Temperature was set to 150 K and maintained using Nosé-Hoover thermostat. Every 25 fs a sample geometry was taken and the electron binding energy was calculated as the difference of the total energy of neutral and negatively charged cluster at different electronic structure levels level for this fixed geometry. Figure S1 shows the distribution of the electron binding energies, together with the experimental measurements for somewhat larger $(H_2O)_{20}^{-}$ clusters by Ma et al at the BMK level.[26] We observe a relatively high overlap between the theoretical calculations and the experiment. Present clusters exhibit slightly larger average ionization energies, indicating somewhat lower susceptibility for the reaction. The difference is larger for the BLYP simulations, suggesting that we underestimate the rate for the CT process.

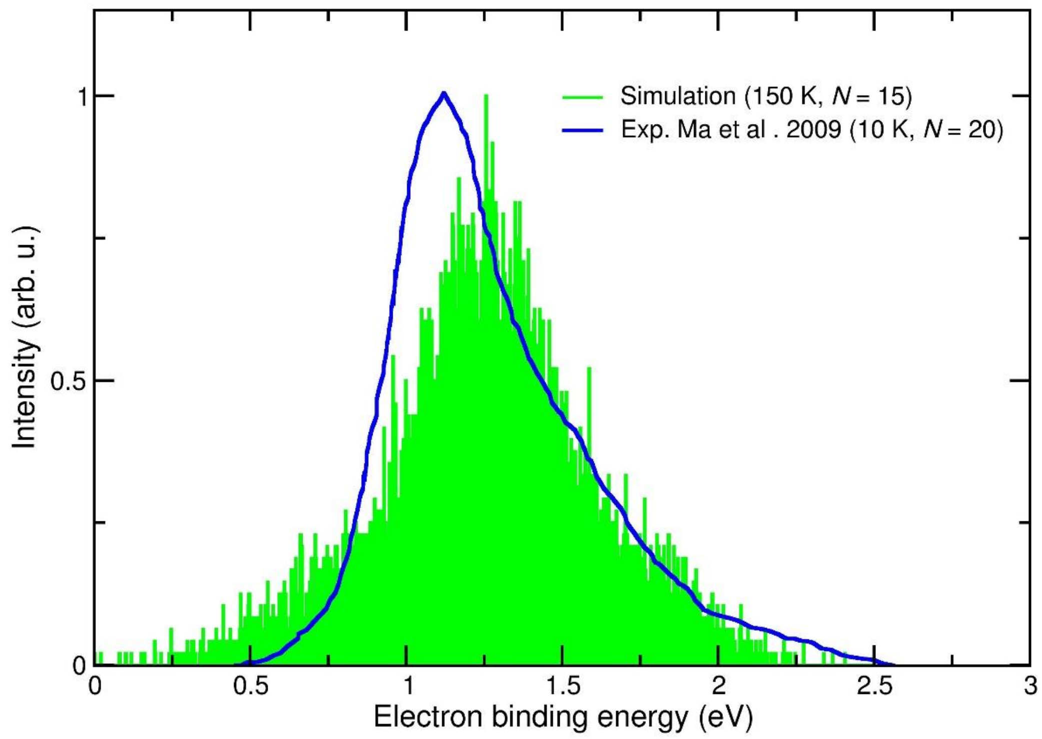

Figure S1. Comparison of vertical detachment energy distributions calculated for anionic water clusters $(H_2O)_{15}^-$ and the experimental data for $(H_2O)_{20}^-$ measured by Ma et al.[26] Distribution of geometries is generated with BLYP/6-31+g* *ab initio* dynamics, the electron detachment energies are recalculated at the BMK/6-31+g* level.

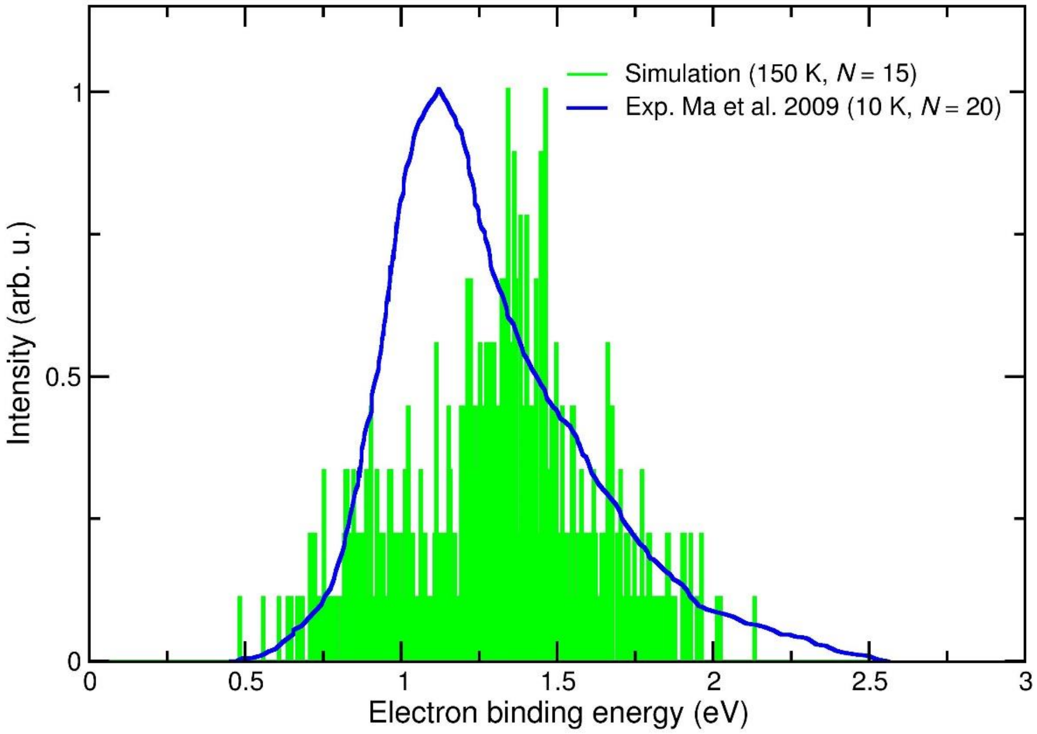

Figure S2. Comparison of vertical detachment energy distributions calculated for anionic water clusters $(H_2O)_{15}^-$ and the experimental data for $(H_2O)_{20}^-$ measured by Ma et al.[26] Distribution of geometries is generated with BLYP/6-31+g* *ab initio* dynamics, the electron detachment energies are recalculated at the BMK/aug-cc-pVDZ level.

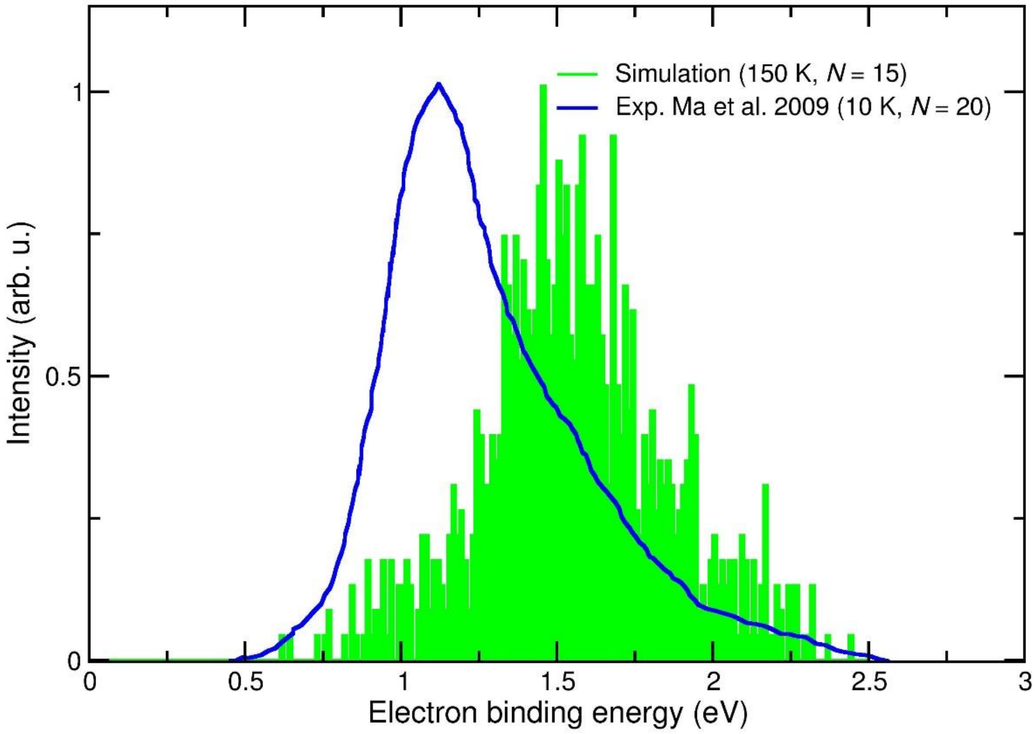

Figure S3. Comparison of vertical detachment energy distributions calculated for anionic water clusters $(H_2O)_{15}^{-}$ and the experimental data for $(H_2O)_{20}^{-}$ measured by Ma et al.[26] Distribution of geometries is generated with BLYP/6-31+g* *ab initio* dynamics, the electron detachment energies are recalculated at the BLYP/6-31+g* level.

b. Vertical electron attachment energy for HNO_3

The structure was optimized at the BMK/aug-cc-pVDZ level and VEAs were recalculated at different levels of theory. The CCSD(T)/aug-cc-pVTZ value corresponds nicely with the experimental value, but this level of theory is computationally extremely demanding and it cannot be used for larger systems. The error of the DFT method is 0.1-0.2 eV. The error partially compensates the DFT error of the VDEs for the water clusters, see above.

	Vertical electron attachment energy / kJ mol^{-1}
BLYP/6-31+g*	−70.71
BLYP/aug-cc-pVDZ	−69.35
BMK/6-31+g*	−76.11
BMK/aug-cc-pVDZ	−67.79
CCSD(T)/aug-cc-pVTZ	−53.71
Experimental energy for electron attachment (ref. 27)	−54.03

7. References

1. C. Berg, T. Schindler, G. Niedner-Schatteburg, and V. E. Bondybey J. Chem. Phys. **102**, 4870 (1995).
2. R. F. Höckendorf, O. P. Balaj, C. van der Linde, and M. K. Beyer Phys. Chem. Chem. Phys. **12**, 3772–3779 (2010).
3. C. S. O'Connor, N. C. Jones, and S. D. Price Int. J. Mass Spectrom. Ion Processes **163**, 131–139 (1997).
4. M. K. Beyer, B. S. Fox, B. M. Reinhard, and V. E. Bondybey J. Chem. Phys. **115**, 9288–9297 (2001).
5. S. T. Arnold, R. A. Morris, and A. A. Viggiano J. Chem. Phys. **103**, 9242 (1995).
6. W. A. Donald, R. D. Leib, J. T. O'Brien, A. I. S. Holm, and E. R. Williams Proc. Natl. Acad. Sci. **105**, 18102–18107 (2008).
7. R. C. Dunbar Mass Spectrom. Rev. **23**, 127–158 (2004).
8. A. V. Titov, I. S. Ufimtsev, N. Luehr, and T. J. Martínez J. Chem. Theory Comput. **9**, 213–221 (2013).
9. I. S. Ufimtsev and T. J. Martínez J. Chem. Theory Comput. **5**, 2619–2628 (2009).
10. C. Lee, W. Yang, and R. G. Parr Phys. Rev. B **37**, 785–789 (1988).
11. A. D. Becke Phys. Rev. A **38**, 3098–3100 (1988).
12. S. Grimme J. Comput. Chem. **27**, 1787–1799 (2006).
13. O. A. Vydrov and G. E. Scuseria J. Chem. Phys. **125**, 234109 (2006).
14. A. D. Boese and J. M. L. Martin J. Chem. Phys. **121**, 3405–3416 (2004).
15. C. Hock, M. Schmidt, R. Kuhnen, C. Bartels, L. Ma, H. Haberland, and B. von Issendorff Phys. Rev. Lett. **103**, 73401 (2009).
16. N. S. Shuman, T. M. Miller, and A. A. Viggiano J. Chem. Phys. **136**, 124307 (2012).
17. D. W. Smith J. Chem. Educ. **54**, 540 (1977).
18. P. Atkins and J. de Paula, *Atkins' Physical Chemistry*, 8th (W. H. Freeman and Company, New York, 2006).
19. H. Shiraishi, G. R. Sunaryo, and K. Ishigure J. Phys. Chem. **98**, 5164–5173 (1994).

20. K. Yagi, Y. Okano, T. Sato, Y. Kawashima, T. Tsuneda, and K. Hirao J. Phys. Chem. A **112**, 9845–9853 (2008).

21. H. M. Lee, P. Tarkeshwar, and K. S. Kim J. Chem. Phys. **121**, 4657–4664 (2004).

22. C. Perez, M. T. Muckle, D. P. Zaleski, N. A. Seifert, B. Temelso, G. C. Shields, Z. Kisiel, and B. H. Pate Science **336**, 897–901 (2012).

23. S. S. Xantheas and T. H. Dunning J. Chem. Phys. **99**, 8774 (1993).

24. E. Flórez, N. Acelas, C. Ibargüen, S. Mondal, J. L. Cabellos, G. Merino, and A. Restrepo RSC Adv. **6**, 71913–71923 (2016).

25. A. Kumar, J. A. Walker, D. M. Bartels, and M. D. Sevilla J. Phys. Chem. A **119**, 9148–9159 (2015).

26. L. Ma, K. Majer, F. Chirot, and B. von Issendorff J. Chem. Phys. **131**, 144303 (2009).

27. J. F. Paulson and F. Dale J. Chem. Phys. **77**, 4006–4008 (1982).
